# Difficulties detecting miRNA-203 in human whole saliva by the use of PCR

**DOI:** 10.4317/medoral.20172

**Published:** 2014-12-05

**Authors:** Martin Lundegard, Karin Nylander, Karin Danielsson

**Affiliations:** 1 Department of Odontology, Umeå University, SE-901 85 Umeå, Sweden; 2 Department of Medical Biosciences, Umeå University, SE-901 85 Umeå, Sweden

## Abstract

Objectives: Oral Lichen Planus (OLP) is a chronic disease of the oral mucosa, and according to the WHO also a pre malignant condition. Micro-RNAs are short non coding RNAs capable of regulating mRNA expression. MiRNA:scan be detected in tissue, blood and human whole saliva (HWS) and recently we have shown miR-203 to be up-regulated in tissue from OLP lesions. Study Design: In order to see whether mRNA as well as miR-203 could be detected also in HWS, saliva from healthy controls and patients with OLP were analysed using two different PCR methods. Results: Results showed low mRNA and miRNA levels in general in HWS samples, making it hard to generate conclusive results. Conclusions: In order to make HWS a valuable source for different analyses, more sensitive PCR techniques capable of detecting very low levels of mRNAand miRNAas well as more efficient methods for extraction of RNA are needed.

** Key words:**miRNA-203, saliva, PCR.

## Introduction

Oral Lichen Planus (OLP) is a chronic disease affecting the oral mucosa, and often also other areas such as the genital mucosa. The prevalence varies between 0.5-2.2% in the normal population, and the disease is most common in middle-aged women. The specific etiology behind OLP is unknown even if the disease shows some typical features of auto immunity (1,2). Clinically the most common forms are reticular and erosive, and symptoms range from none to almost unbearable pain (3). OLP is by the World Health Organization, WHO, classified as a potentially malignant condition with higher risk of developing into squamous cell carcinoma, SCC. The potential for malignant development has, however, been widely questioned (4).

Human whole saliva, HWS, consists mainly of water and a broad spectrum of proteins, nucleic acids, electrolytes and hormones. Its composition and regulation is complex, depending on constantly varying exogenous and endogenous factors such as overall health of the individual, drugs, hormonal status, age, heredity and oral hygiene (5). HWS is mainly secreted from the major glands, the parotid, sublingual and submandibular, and only around 10% comes from the accessory glands. Salivary secretion can be both basal and stimulated, the latter generating a volume of 500-1000 ml per day (6). No significant difference in either composition or volume has been seen between genders, whereas a reduced basal secretion volume has been shown with age (7). As HWS can be easily collected through a non invasive technique (7) its potential use in different analyses has been under investigation in several studies.

MicroRNAs (miRNAs) are short, 22nt long, non-coding RNAs present in tissue, blood and HWS. They regulate messenger-RNAs (mRNAs) resulting in repression or inhibition of proteins (8). A single miRNA can target multiple mRNAs enabling it to simultaneously affect several pathways, and many miRNAs have shown to be important in carcinogenesis and the tumorigenic process (9). A study of patients with oral carcinoma showed significantly higher expression of microRNA-31, miR-31, in saliva from tumour patients compared to a control group (9). Looking at tissue samples, we have shown another micro-RNA, miR-203, which is expressed only in keratinocytes (10,11), to be over expressed in OLP lesions compared to healthy controls (12). Based on these results, we now wanted to investigate if this over expression also could be seenin HWS from patients with OLP compared to healthy controls.

## Material and Methods

-Patient material and collecting process

**Figure 1:**
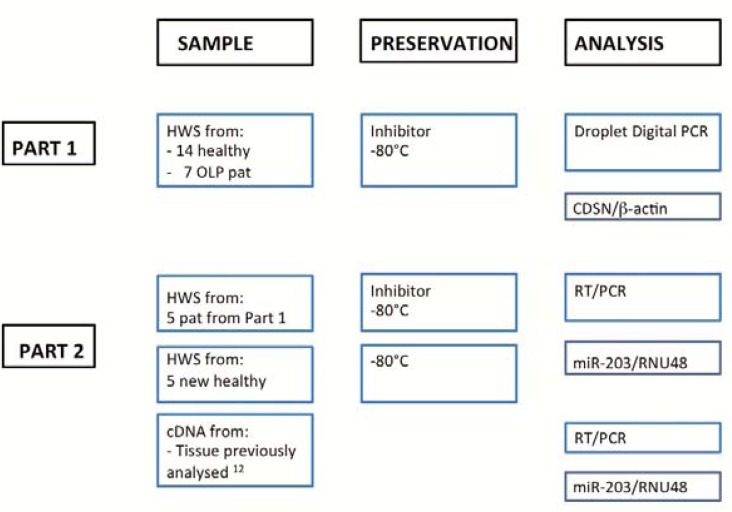
General workflow displaying outline for collection and handling of samples and analyses performed.

The study was approved by the Ethical Committee at Umeå University, Dnr 2011-252-32M, and all participants had been given information both orally and in writing. The inclusion criteria for the controls were: age 18 or above, no drugs affecting salivary secretion and no systemic disease. Saliva was also collected from consecutive patients referred to the specialist clinic at the Department of Odontology, Umeå University, clinically and histologic ally diagnosed with OLP. Samples were collected using a stimulated saliva test (6), and participants unable to provide 2ml of saliva during the given five-minute time period were excluded. Samples from a total of 21 individuals were collected, 14 healthy individuals and 7 diagnosed with OLP. The HWS samples were divided into three 200µl aliquots, each directly mixed with 1ml of Saliva degradation inhibitor (Saliva Incubation Mix, Qiagen Germany) and kept at room temperature for 1h before freezing and storage at -80°C, shown to be most efficient in prevention of degradation (7). From each participant there were thus three samples available for further analysis. The collection and handling process is summarized in figure 1, Part 1.

In order to determine if addition of saliva inhibitor could interfere with the PCR reactions 5 of the samples from Part 1 were compared with 5 new HWS samples from healthy individuals without addition of any saliva inhibitor. As a control for the PCR reaction 5 cDNA samples from tissue previously analyzed for expression of miR-203 (12) were included (Fig. 1, Part 2).

-PCR Analyses

To start with, we wanted to see if RNA was detectable at all in our HWS-samples, and thus the QX100™ Droplet Digital™ PCR (ddPCR™) system, was chosen due to its capability to detect very low levels of RNA (>66ng/20µL/sample). Primers for corneodesmosin (CDSN) which previously has been shown to be highly up regulated in LP-lesions (13) and β-Actin as reference were chosen for analysis (14). In the first run FaDu cells (hypo pharyngeal carcinoma; ATCC) and HEKa cells (Human epidermal kerationcytes, Life Technologies, USA) were used as positive controls.

In the second part the miRCURYLNA™ Universal RT microRNA PCR kit (Exiqon, Denmark) was used for detection of miRNA, consisting of first strand cDNA synthesis and real time PCR amplification. Quantitative RT-PCR for miRNAs was performed with IQ5 multicolor real-time PCR detection system (Bio-Rad laboratories Inc., USA) 

Samples from all three groups in Part 2 were analyzed simultaneously using primers for miRNA-203 and RNU48, also called 48u, as a reference gene (15). All samples were tested in duplicate and a requirement for detection in both sub samples was set as inclusion criteria in order to be considered positive.

-RNA extraction and cDNA preparation

The RNeasy Micro Kit (Qiagen, Hilden, Germany) was used for extraction of RNA. Samples were thereafter analyzed for RNA levels and quality using the Nano Drop-system (Nano Drop ND-1000 Spectrophotometer, Nano Drop v 3.1.2 software). The quality control criteria, based upon the Nano Drop 100 Technical & Bulletin, with an RNA content of >3ng/μL and a 260/280value of 1.80-2.00 was applied.

As two different PCR methods were used, cDNA synthesis was accordingly performed in two different ways. For samples in Part 1, using the QX100 Droplet Digital PCR ddPCR™ (BioRad, California, USA) the cDNA-kit “Droplet PCR Supermix” (BioRad, California, USA) was used, and for samples in Part 2 analyzed by Bio-Rad iQ5 Real Time PCR Detection System (BioRad, California, USA) the cDNA kit included in ExiqonmiRCURY LNA Universal RT microRNA PCR - kit (Exiqion, Copenhagen, Denmark) was used. Samples were kept on ice during the whole process and all samples were analyzed in duplicate during the PCR process. For samples in Part 2, RNA-extraction, Nano Drop measurement and cDNA synthesis was performed simultaneously for the HWS samples, whereas cDNA already was available from tissue samples (12).

-Statistical Analysis

All data were converted to Microsoft Excel for further analysis and graph formation, except data collected from Part 1, and analyzed using the “QuantaSoft v 1.4.0”.

## Results

-RNA extraction and quality

RNA was extracted at two separate occasions, A and B, from each of the 21 HWS samples in Part 1, giving a total of 42 RNA extracts. However, as shown in  Table 1 only 2 out of the 42 samples met the quality control criteria (5A and 24A). Twelve of the 42 samples had a negative ng/µL-value and three a negative 260/280-value.

**Table 1:**
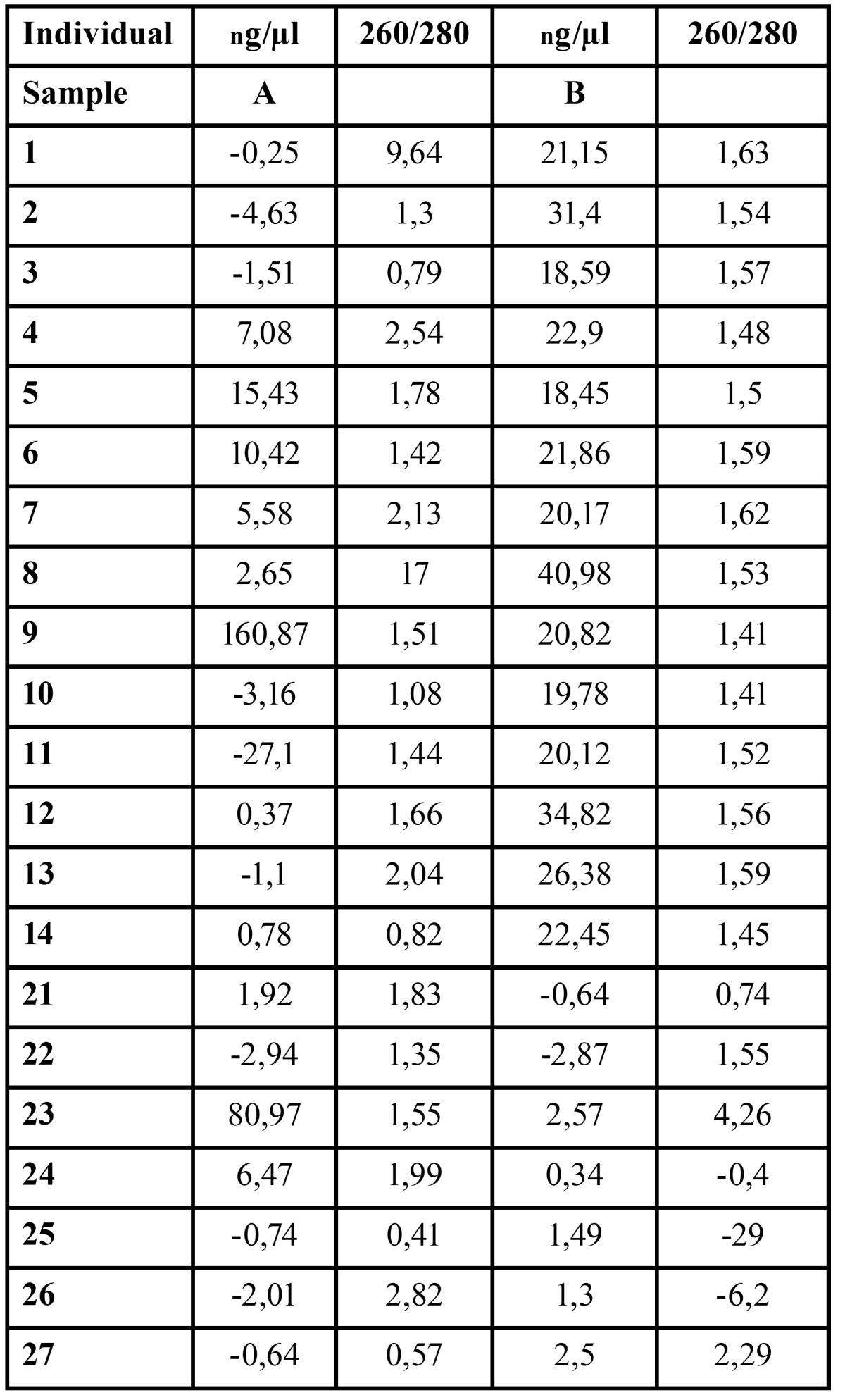
Quality control. Only two samples (5 and 24) fulfilled the quality control based on the NanoDrop100 Technical Bulletin with an RNA content of > 3ng/μL and a 260/280 value of 1.80-2.00.

-Droplet Digital™ PCR (ddPCR™)

Corneodesmosin, CDSN, could only be detected in HEKa cells, whereas β-actin was detected in one HWS sample, 5A, but not in its duplicate 5B. Looking at quality control, the 5A sample met the quality control criteria for the NanoDrop whereas 5B did not.

-Bio-Rad iQ5 (RT/PCR)

**Figure 2:**
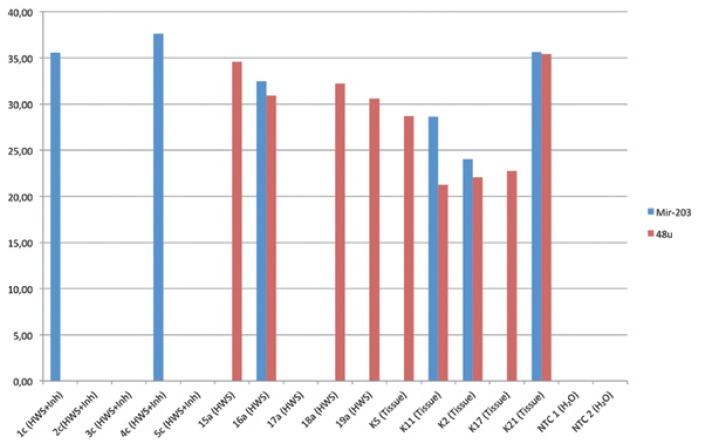
Results from Part 2 displaying results (CƬ-Mean) from the RT/PCR analysis of miR-203 and the refe-rence gene 48u.

As shown in figure 2 both miR-203 and the reference gene 48u could be detectedin 4 out of the 15 analyzedsamples. Three of these were tissue samples, previously analyzed (12), and now included as controlsfor the PCR reaction, and one was aHWS sample from Part 2, collected without addition of saliva inhibitor. MiR-203 was detected in duplicate in 6 out of 15 samples, and the reference gene 48u in 9 out of 15 samples.

## Discussion

The possibility to use HWS in different analyses is very attractive, mainly due to the non invasive way of collecting samples. A problem can, however, be levels ofRNA, which in our HWS samples were low compared to previous studies reporting 5-10 times higher values (7,16). These studies did not either report samples with negative values, whereas almost a third, 29%, of our samples showed negative values, in our experience representing values too low to be measured. Comparing protocols between the studies showed that Patel *et al*. (7) used a different kit for collection of saliva and extraction of RNA while Pandit and coworkers (16) had used a modified extraction method with the addition of isopropanol alcohol/chloroform without addition of a saliva inhibitor. The addition of isopropanol alcohol and chloroform in the extraction process aids in removing proteins, and at least theoretically, the extracts used in their study could thus be more “pure RNA extracts” compared to ours. Furthermore, their samples were constantly kept at +4°C, whereas we in our experiment followed the protocol from the manufacturer and kept samples at room temperature after the addition of saliva inhibitor. Temperature changes may inflict on RNA degradation in HWS during the extraction process. The sensitivity of Nano Drop measurements must also be addressed based on the inconsistency seen when repeating measurement of the same sample. Using Nano Drop only 1µL of the sample is needed, and increasing the volume to 2µL did not give more consistent results. We chose to include a standard amount of 10µL from each samplein the cDNA synthesis process, irrespective of the measured values.

The fact that our samples originated from people of different ethnic origin is less likely to explain the difference in results, as is gender (14).

The Digital Droplet, dd, PCR technique is based on creation and classification on droplets without any amplification. When analyzing our results, it must be noticed that all control samples from cell lines showed positive results, supporting that the method was working properly. In the HWS samples levels of β-actin and CDSN were, however, most likely too low to be detected, even when using the sensitive ddPCR method.

Strangely enough, the RT/PCR method which is less sensitive in detection of low levels of RNAgavemore positive results. Of the 15 samples analyzed in Part 2, both miR-203 and 48u could be detected in duplicate in three tissue samples previously analyzed (12) and one HWS sample where no saliva inhibitor had been added. In another HWS sample without inhibitor the reference gene 48u was detected, and in two of the 5 HWS samples with inhibitor miR-203 could be detected. Based on this limited material it is, however, not possible to judge whether addition of saliva inhibitor has any impact on the possibility to detect RNA and miRNA in HWS samples.

In summary, we were in our material not able to detect RNA and miRNA in most of our HWS samples by the use of two different PCR-methods. This was most likely due to extremely low levels of RNA present in HWS compared to tissue samples. In order to successfully use HWS in future analyses there is therefore a need for more efficient methods for RNA extraction and more sensitive techniques for analyses. Alternatively pre-amplification could be considered in analysis of these samples (17).
